# Uma Constelação de Acidente Vascular Cerebral e Hipoxemia após Extração do Eletrodo do CDI: Papel do Forame Oval Patente

**DOI:** 10.36660/abc.20240303

**Published:** 2024-09-17

**Authors:** Ashish H. Shah, Petra Jenkins, Heiko Schneider

**Affiliations:** 1 University of Manitoba St. Boniface Hospital Section of Cardiology Manitoba Canadá Section of Cardiology, St. Boniface Hospital, University of Manitoba, Manitoba – Canadá; 2 Liverpool Heart and Chest Hospital Liverpool Inglaterra Liverpool Heart and Chest Hospital, Liverpool – Inglaterra; 3 University of Zurich University Hospital Zurich Department of Cardiology Zurich Suíça Department of Cardiology, University Hospital Zurich, University of Zurich, Zurich – Suíça

**Keywords:** Forame Oval Patente, Acidente Vascular Cerebral, Hipóxia

Uma mulher de 78 anos com cardiomiopatia isquêmica conhecida foi submetida eletivamente à extração do eletrodo do cardioversor desfibrilador implantável (CDI) e ao implante de um novo eletrodo. Duas horas após o procedimento, apresentou disfasia expressiva e hemiplegia direita, o que foi confirmado como resultado de um acidente vascular cerebral isquêmico em uma tomografia computadorizada de crânio. Além disso, a paciente desenvolveu hipoxemia em repouso com saturação de oxigênio de 87-88% em ar ambiente, que melhorou apenas parcialmente com oxigênio de alto fluxo.

Uma investigação mais aprofundada revelou um shunt da direita para a esquerda através de um forame oval patente (FOP) em um ecocardiograma transtorácico (ETT). Exames ETT anteriores realizados nos últimos 5 anos não mostraram qualquer patologia valvular ou shunt da direita para a esquerda através do FOP. O paciente estava em terapia anticoagulante oral direta para fibrilação atrial, que foi interrompida eletivamente 2 dias antes do procedimento, e foi iniciada heparina de baixo peso molecular como terapia ponte.

Suspeitou-se que o acidente vascular cerebral fosse causado pela embolização de um coágulo sanguíneo ou fibrina do eletrodo extraído do CDI, uma complicação conhecida do procedimento. Considerando que se pensava que a hipoxemia se devia à distorção da estrutura cardíaca, particularmente à relação entre a veia cava superior (VCS) e a veia cava inferior (VCI), o que pode resultar na formação alterada de vórtices no átrio direito e facilitar shunt da direita para a esquerda (Painel A; Vídeo 1).^1^

**Figura 1:**
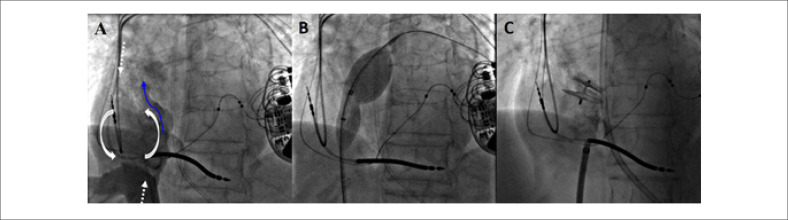
Fechamento transcateter do forame oval patente tratando hipóxia mediada por shunt da direita para a esquerda. Painel A) Angiografia do átrio direito demonstrando shunt da direita para a esquerda através do forame oval patente; Painel B) oclusão por balão do forame oval patente; Painel C) fechamento transcateter do FOP. A seta branca tracejada descreve o fluxo na VCS e na VCI; a seta curva descreve o fluxo no átrio direito e a seta azul descreve o fluxo através do FOP.

Como a paciente permaneceu persistentemente hipoxêmica devido ao shunt da direita para a esquerda mediado pelo FOP e estava acamada devido ao acidente vascular cerebral, foi submetida à intervenção transcateter. A pressão média do átrio direito foi de 8 mmHg, enquanto a pressão média do átrio esquerdo foi de 11 mmHg. A angiografia do átrio direito confirmou o shunt de sangue para o átrio esquerdo através do FOP (Figura 1A; Vídeo 1). A saturação venosa pulmonar bilateral de oxigênio foi de 96-97%, enquanto a saturação aórtica foi de 88%, confirmando que a hipoxemia era devida ao shunt direita-esquerda. Saturação sistêmica normalizada após oclusão do FOP por balão (Figura 1B; Vídeo 1). O paciente foi então submetido ao fechamento transcateter do FOP com dispositivo oclusor Amplatzer^®^ de 25 mm. Um vazamento residual através do FOP foi observado após a implantação do dispositivo, no entanto, a saturação sistêmica de oxigênio permaneceu entre 94-95% em ar ambiente (Figura 1C; Vídeo 2). Especialmente entre pacientes submetidos ao fechamento transcateter do FOP tratando shunt da direita para a esquerda, nossa publicação anterior descreveu (1) vazamento residual frequente, (2) normalização da hipoxemia sistêmica apesar do vazamento residual, (3) necessidade frequente de uso de dispositivo não FOP e (4) posição horizontal do dispositivo, provavelmente corroborando com anatomia distorcida do septo interatrial.^2^

**Vídeo 1:**
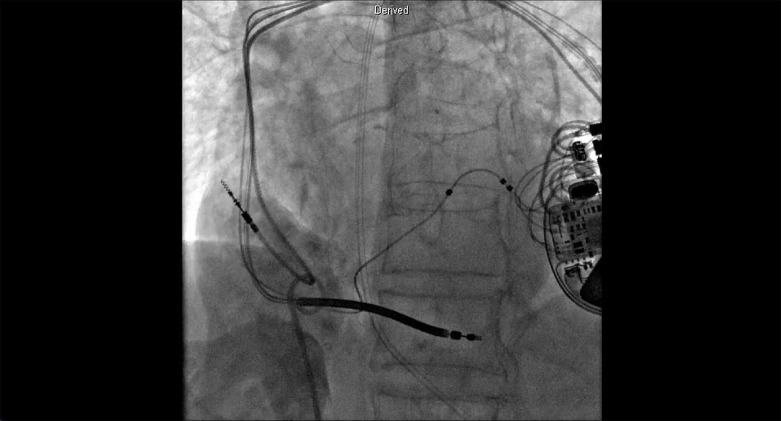
Angiografia do átrio direito demonstrando shunt da direita para a esquerda. Link: http://abccardiol.org/supplementary-material/2024/12108/2024-0303_video_01.mp4

**Vídeo 2:**
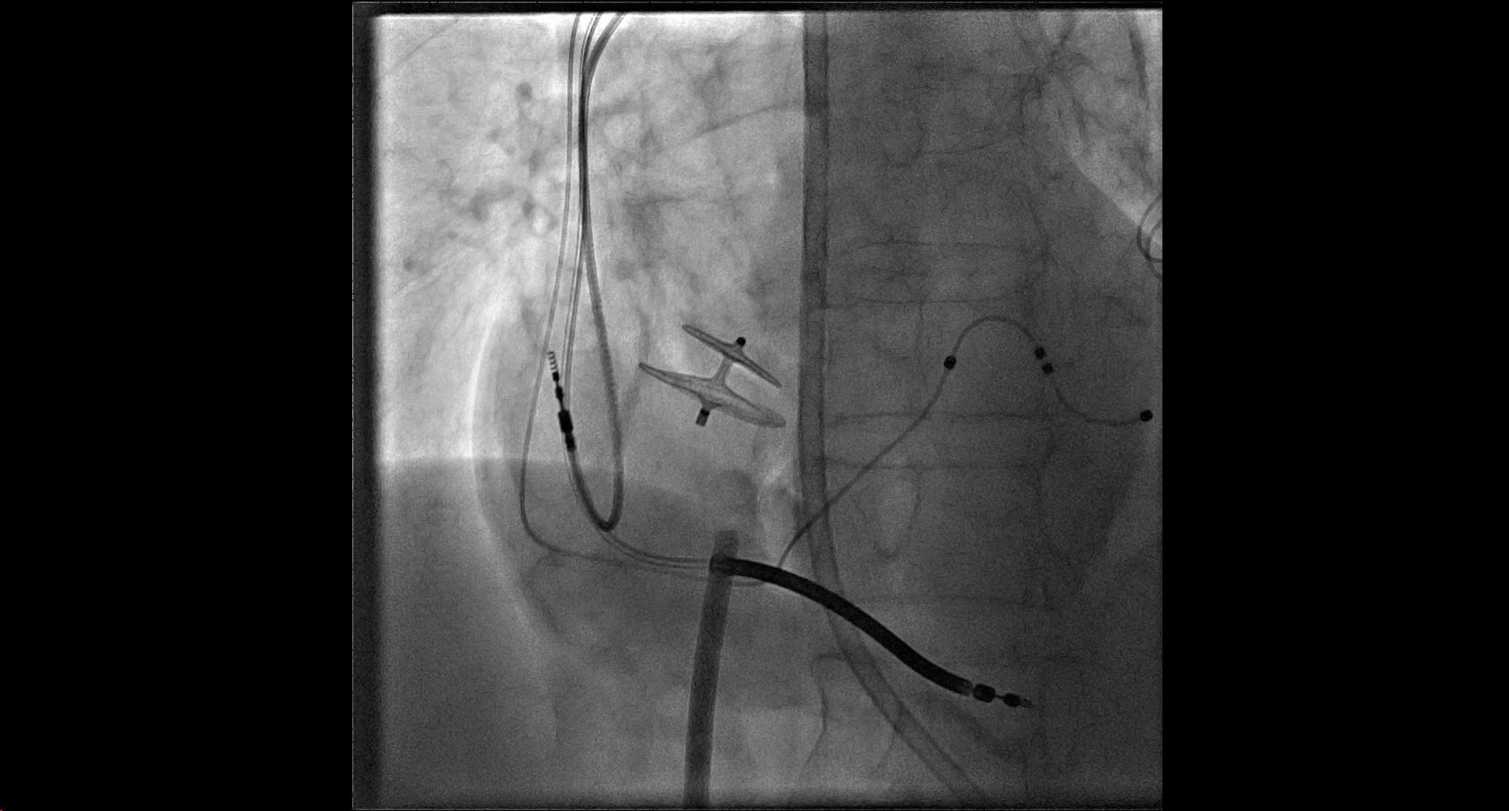
Fechamento transcateter do forame oval patente com shunt residual por meio do dispositivo Amplatzer® de fechamento de FOP. Link: http://abccardiol.org/supplementary-material/2024/12108/2024-0303_video_02.mp4

Entre pacientes com dispositivos cardíacos implantáveis, a presença de FOP é um fator de risco independente para acidente vascular cerebral.^3^ O caso destaca uma ocorrência rara de shunt de sangue da direita para a esquerda mediado por FOP e seu conteúdo após a extração do eletrodo do CDI, que geralmente é observado após cirurgias toraco-abdominais.^2^ Os médicos responsáveis pelo tratamento devem estar cientes de tais patologias associadas ao FOP, uma vez que a intervenção transcateter pode eliminar eficazmente o shunt da direita para a esquerda mediado pelo FOP, bem como a embolização sistémica.
