# Supplementary material for: Uma Constelação de Acidente Vascular Cerebral e Hipoxemia após Extração do Eletrodo do CDI: Papel do Forame Oval Patente
Source: Arq Bras Cardiol. 2024 Sep 17;121(8):e20240303. [Article in Portuguese] doi: 10.36660/abc.20240303 (PMC11495801; doi:10.36660/abc.20240303)
Supplement: Translation

# Translation: A Constellation of Stroke and Hypoxemia Post ICD Lead Extraction: Role of Patent Foramen Ovale

**DOI:** 10.36660/abc.20240303i

**Authors:** Ashish H. Shah, Petra Jenkins, Heiko Schneider

**Affiliations:** 1 Manitoba Canada Section of Cardiology, St. Boniface Hospital, University of Manitoba, Manitoba – Canada; 2 Liverpool UK Liverpool Heart and Chest Hospital, Liverpool – UK; 3 Zurich Switzerland Department of Cardiology, University Hospital Zurich, University of Zurich, Zurich – Switzerland

**Keywords:** Patent Foramen Ovale, Stroke, Hypoxia

A 78-year-old woman with known ischemic cardiomyopathy underwent elective implantable cardioverter defibrillator (ICD) lead extraction and a new lead implantation. Within 2 hours post-procedure, she experienced expressive dysphasia and right hemiplegia, which was confirmed to be due to an ischemic stroke on a brain CT scan. Additionally, the patient developed resting hypoxemia with oxygen saturation of 87-88% on room air that only partially improved with high-flow oxygen.

Further investigation revealed a right-to-left shunt through a patent foramen ovale (PFO) on a trans-thoracic echocardiogram (TTE). Previous TTE examinations over the past 5 years did not show any valvular pathology or right-to-left shunt through the PFO. The patient was on direct oral anticoagulant therapy for atrial fibrillation, which was electively stopped 2 days before the procedure, and low molecular weight heparin was initiated as a bridging therapy.

The stroke was suspected to be caused by embolization of a blood clot or fibrin from the extracted ICD lead, a known procedural complication. Whereas hypoxemia was thought to be due to distortion of cardiac structure, particularly the relationship between the superior vena cava (SVC) and inferior vena cava (IVC), which can result in altered vortex formation in the right atrium and facilitate right-to-left shunting (Panel A; Video 1).^1^

As the patient remained persistently hypoxemic due to the PFO-mediated right-to-left shunt and was bedbound due to the stroke, she underwent transcatheter intervention. The right atrial mean pressure was 8 mmHg, whereas the left atrial mean pressure was 11 mmHg. The right atrial angiogram confirmed the shunting of blood to the left atrium through the PFO (Figure 1A; Video 1). Bilateral pulmonary venous oxygen saturation was 96-97%, while aortic saturation was 88%, confirming the hypoxemia was due to the right-to-left shunt. Systemic saturation normalized after balloon occlusion of the PFO (Figure 1B; Video 1). The patient then underwent transcatheter closure of the PFO using an Amplatzer® 25 mm PFO occluder device. A residual leak through the PFO was observed after device deployment, however, her systemic oxygen saturation remained 94-95% on room air (Figure 1C; Video 2). Especially among patients undergoing transcatheter closure of PFO treating right-to-left shunt, our previous publication described (1) frequent residual leak, (2) normalization of systemic hypoxemia despite the residual leak, (3) frequent need for non-PFO device use, and (4) horizontal device position, likely corroborating with distorted interatrial septal anatomy.^2^

**Figure 1:** Transcatheter closure of patent foramen ovale treating right to left shunt mediated hypoxia. Panel A) Right atrial angiogram demonstrating right to left shunting through patent foramen ovale; Panel B) balloon occlusion of patent foramen ovale; Panel C) transcatheter PFO closure. The white broken arrow describes the flow in SVC and IVC; the curved arrow describes the flow in the right atrium and the blue arrow describes the flow through PFO.

**Video 1:** Right atrial angiogram demonstrating right to left shunt. Link: http://abccardiol.org/supplementary-material/2024/12108/2024-0303_video_01.mp4

**Video 2:** Transcatheter closure of patent foramen ovale with residual shunt through the Amplatzer® PFO closure device. Link: http://abccardiol.org/supplementary-material/2024/12108/2024-0303_video_02.mp4

Among patients with implantable cardiac devices, the presence of PFO is an independent risk factor for stroke.^3^ The case highlights a rare occurrence of PFO-mediated right-to-left shunting of blood and its content post-ICD lead extraction that is usually seen after thoraco-abdominal surgeries.^2^ Treating physicians should be cognizant of such PFO-associated pathologies, as transcatheter intervention can effectively eliminate PFO-mediated right-to-left shunt as well as systemic embolization.
